# Impact of hyperparathyroidism and its different subtypes on long term graft outcome: a single Transplant Center cohort study

**DOI:** 10.3389/fmed.2023.1221086

**Published:** 2023-08-10

**Authors:** Paolo Molinari, Anna Regalia, Alessandro Leoni, Mariarosaria Campise, Donata Cresseri, Elisa Cicero, Simone Vettoretti, Luca Nardelli, Emilietta Brigati, Evaldo Favi, Piergiorgio Messa, Giuseppe Castellano, Carlo M. Alfieri

**Affiliations:** ^1^Unit of Nephrology, Dialysis and Kidney Transplantation, Fondazione IRCCS Ca’ Granda Ospedale Maggiore Policlinico di Milano, Milan, Italy; ^2^Post-Graduate School of Specialization in Nephrology, University of Milano, Milan, Italy; ^3^Department of Clinical Sciences and Community Health, Università degli Studi di Milano, Milan, Italy; ^4^General Surgery and Kidney Transplantation, Fondazione IRCCS Ca' Granda Ospedale Maggiore Policlinico, Milan, Italy; ^5^Department of Nephrology, Dialysis and Kidney Transplants, IRCCS Ca’ Granda Foundation Maggiore Policlinico Hospital, Milan, Italy

**Keywords:** kidney transplantation, parathormone, hyperparathyroidism, tertiary hyperparathyroidism, graft outcome

## Abstract

**Purpose:**

We studied the association between parathormone (PTH) levels and long-term graft loss in RTx patients (RTx-p).

**Methods:**

We retrospectively evaluated 871 RTx-p, transplanted in our unit from Jan-2004 to Dec-2020 assessing renal function and mineral metabolism parameters at 1, 6, and 12 months after RTx. Graft loss and death with functioning graft during follow-up (FU, 8.3[5.4–11.4] years) were checked.

**Results:**

At month-1, 79% had HPT, of which 63% with secondary HPT (SHPT) and 16% tertiary HPT (THPT); at month-6, HPT prevalence was 80% of which SHPT 64% and THPT 16%; at month-12 HPT prevalence was 77% of which SHPT 62% and THPT 15%. A strong significant correlation was found between HPT type, PTH levels and graft loss at every time point. Mean PTH exposure remained strongly and independently associated to long term graft loss (OR 3.1 [1.4–7.1], *p* = 0.008). THPT was independently associated with graft loss at month-1 when compared to HPT absence and at every time point when compared to SHPT. No correlation was found with RTx-p death. Discriminatory analyses identified the best mean PTH cut-off to predict long-term graft loss to be between 88.6 and 89.9 pg/mL (AUC = 0.658). Cox regression analyses highlighted that THPT was strongly associated with shorter long-term graft survival at every time-point considered.

**Conclusion:**

High PTH levels during 1st year of RTx seem to be associated with long term graft loss.

## Introduction

Mineral and bone disorders (MBD) are frequent from the early stages of CKD and strongly influence the morbidity and mortality of CKD patients. Even in the presence of an RTx, some mineral metabolism (MM) alterations can persist, also in the presence of complete renal function recovery and are responsible for post-renal transplant MBD (post-RTx-MBD) (1).

In around 60% of cases, even in the presence of a well-functioning graft, a persistent increase of PTH can be seen (2–7). The actual pathophysiological mechanism is yet to be clearly defined, even if dialysis vintage, pre-RTx calcimimetic use, pre-RTx PTH levels >300, obesity and hypercalcemia at RTx seems to be the main risk factors in post RTx hyperparathyroidism (HPT) development (7–11).

The impact of persistent HPT on graft outcome is still debated. In some studies HPT after RTx was associated with graft loss and mortality (8–12). Some other evidence pointed out that post-RTx HPT increases the risk for kidney stones formation, graft disfunction and cognitive impairment (13). Anyways, the optimal PTH value to achieve in RTx-p is still to be defined, and precise guidelines about post-RTx HPT monitoring and management are lacking (14). On the contrary, there are also some works in the literature that did not find a relationship between post-RTx HPT and long-term graft outcome (8, 15).

This issue of secondary and tertiary HPT in RTx has been reviewed by our group (16, 17). To follow-up that initial work, we performed a retrospective observational cohort study to better understand the potential role of HPT in our cohort of RTx patients (RTX-p).

## Materials and methods

### Study design and characteristics

We performed a retrospective cohort study including 871 RTx-p transplanted and followed in our Department (Unit of Nephrology, Dialysis and Kidney Transplantation, Fondazione IRCCS Ca' Granda Ospedale Maggiore Policlinico di Milano, Milan, Italy) from January 2004 to December 2020. Patients that did not manage to achieve 1 year of FU were excluded from the study; in particular, the global characteristics of these patients did not siginifically differ from included and studied patients. At RTx, for each of the 871 RTx-p examined, anamnestic and anthropometric characteristics were recorded. Urinary and blood evaluations were performed after 12 h of fasting at the Transplant Clinic of Our Department and analyzed by the Central Laboratory of our Hospital. Clinical and biochemical data were then recorded at 1st (T1) and 12th (T12) month after RTx. In particular, the following data regarding MBD were considered: alkaline phosphatase (ALP), serum calcium, 25-OH Vitamin D and 1,25-OH Vitamin-D. Mean (T1-T6-T12) PTH level was also considered in statistical analyses.

All data used were derived from our dataset, only if complete data collection was available. All incomplete data were not considered in our analyses.

### Biochemical analyses

For iPTH detection and quantification, we used the electro-chemiluminescence immune-assay (ECLIA) and the E170 module for the Modular Analytics (Roche Diagnostics, Basel, Switzerland), whereas serum vitamin D levels were assessed using an enzyme immune-assay (Kit EIA AC-57 FI, Immuno-Diagnostic System, Boldon, United Kingdom) with highly specific 25-hydroxivitamin D sheep antibody and enzyme-labeled avidin (horseradish peroxidase). Vitamin D status was classified as follows: deficiency if 25-hydroxivitamin D < 20 ng/mL, insufficient if 25-hydroxivitamin D 20–30 ng/mL, sufficient if 25-hydroxivitamin D > 30 nog/ml. Normality range for iPTH was 6.5–36.8 pg/mL.

All the other biochemical parameters were measured according to the routine methodology used in our central laboratory. The sCr assessed was done by Jaffe’s reaction, whereas eGFR was estimated using the Modification of Diet in Renal Disease (MDRD) formula (18, 19). Urinary protein excretion was assessed by measuring the 24-h urinary collection protein through the immunoturbidimetric method.

### HPT definition

HPT was defined by PTH levels above 36.8 pg/mL. HPT type was further defined by corrected serum calcium level. In particular SHPT was defined as PTH above 36.8 pg/mL and corrected serum Ca under 10.4 mg/dL. THPT, instead, was defined as PTH above 36.8 pg/mL and corrected serum Ca above 10.4 mg/dL. Corrected calcium levels (Cca) were calculated according to the following formula Cca = calcium level + (40 − albuminemia)/40.

### Immunosuppressive therapy and MBD related therapies

According to our internal protocol, the immune suppressive induction therapy was composed by Basiliximab or ATG according to the characteristics of the donor and recipient.

Almost all patients were treated with standard maintenance immunosuppression after induction (e.g., CNI, mycophenolate/mycophenolic acid [MMF/MPA], steroids), as per international guidelines and Center protocols.

Regarding MBD therapies, most patients with HPT were left untreated, unless PTH values exceeded 2.5 times normal values. This approach did not change significantly during the years. Mineral metabolism anomalies were treated according to the clinical protocols of our Center (16).

### Outcomes and follow up

Patients were followed up from Jan 2004 to Dec 2020 for a median time of 8.3[5.4–11.4] years.

At the end of follow up (FU), the following outcomes were evaluated: Graft loss (GL), defined by the need to restart dialysis; RTx-p death with functioning graft.

### Statistical analyses

Continuous variables were expressed as median [25–75% ile]. Differences between the groups were determined using the Student’s *t*-test, the Wilcoxon-Mann–Whitney test, Kruskal Walls, ANOVA, the Chi square test, and the Fisher test when indicated. Linear regression analyses model was used to determine the association between baseline biochemical and anthropometric parameters and PTH levels at different time points. Logistic regression models were performed for multivariate analyses. ROC curve was used to determine the best cut-off value for the variables examined. Survival analyses were performed using KM curves and statistical differences were evaluated with COX regression analysis. Statistical significance was set for *p* < 0.05 values. Statistical analyses were performed using Statistica^®^ version 10 and SPSS 26^®^.

### Ethical approval and informed consent

Treatments and procedures herein reported were in accordance with the ethical standards of the institutional committee at which it was conducted (Fondazione IRCCS Ca' Granda Ospedale Maggiore Policlinico Ethical Committee, Protocol ID 4759-1837/19), as well as with the 1964 Helsinki declaration and its later amendments, or comparable ethical standards. Given the observational and retrospective nature of the study, it was not necessary to obtain informed consent from patients. In any case, all the data were collected digitally, analyzed and reported in the results in a totally and anonymous manner.

## Results

### Anthropometric and baseline cohort characteristics

As depicted in Table 1 of the overall cohort composed of 871 RTx-p, the majority were male (505, 58%). The cohort had a mean age at RTx of 49 ± 12. Most of RTx-p were subjected to hemodialysis (HD) before RTx, and 17% was transplanted from a living donor. Delayed graft function (DGF) defined as the need for dialysis during the first week after RTx was found in 15%.

**Table 1:** Anthropometric, pre RTx and baseline characteristics of the cohort.

Variables	Overall cohort (*n* = 871)
Age at RTx (years)	49 ± 12.8
Dialysis vintage (months)	53.5 ± 52
Cold ischemia time (hours)	15 ± 12.4
Blood systolic pressure (mmHg)	
*T1*	130 ± 16
*T12*	129 ± 16
Blood dyastolic pressure (mmHg)	
*T1*	79 ± 10
*T12*	79 ± 10
BMI	
*T1*	23.4 ± 3.8
*T12*	24.3 ± 3.8
Gender	
*M*	505 (58)
*F*	366 (42)
Dialysis type	
*HD*	618 (71)
*PD*	183 (21)
*Pre-emptive*	70 (8)
Transplant donor	
*Deceased*	727 (83)
*Living*	144 (17)
Diabetes pre-RTx	
*Yes*	669 (77)
*No*	202 (23)
DGF	
*Yes*	131 (15)
*No*	740 (85)
Hyperparathyroidism T1	
*No*	183 (21)
*Secondary*	549 (63)
*Tertiary*	139 (16)
Hyperparathyroidism T6	
*No*	180 (20)
*Secondary*	552 (64)
*Tertiary*	139 (16)
Hyperparathyroidism T12	
*No*	182 (23)
*Secondary*	541 (62)
*Tertiary*	131 (15)
Vitamin D status T1	
Deficient	697 (80)
Insufficient	148 (17)
Sufficient	31 (4)
Vitamin D status T6	
Deficient	547 (71)
Insufficient	157 (18)
Sufficient	96 (11)
Vitamin D status T12	
Deficient	522 (60)
Insufficient	218 (25)
Sufficient	131 (15)
Rejection episodes (1 or more)	
*1 yr*	548 (63)
*After 1 yr*	627 (72)
Graft loss	
*Yes*	105 (12)
*No*	766 (88)
Death	
*Yes*	113 (13)
*No*	758 (87)

RTx, renal transplantation; HD, hemodialysis; PD, peritoneal dialysis; DGF, delayed graft function; BMI, body mass index. Numerosity is reported as number (percentage) while anthropometric values are listed as mean ± standard deviation.

Hyperparathyroidism (HPT) was highly prevalent in our cohort during the first year of RTx, ranging from 77 to 80%. In particular, secondary hyperparathyroidism (SHPT) was the main type of HPT (around 62–64% of the total cohort), while tertiary hyperparathyroidism (THPT) assessed around values of 15–16% of RTx-p.

Graft rejection had a consistent prevalence in our cohort. Indeed, 62% of RTx-p experienced at least 1 rejection episode during the first year; this percentage further increased when considering graft rejection episodes after the first year, assessing around 72% of RTx-p. At the end of FU, 12% of RTx-p underwent graft loss, while 13% of them died. These percentages did not vary significantly in patients transplanted from a deceased donor (13.5% of GL and 13% of deaths) while was markedly lower in patients who received an organ from a living donor (8.5% of GL and 7% of deaths respectively).

In Supplementary Table S1 data regarding therapies aimed at controlling MBD in our cohort are listed. Of note, Cinacalcet use before RTx was found only in 5% of RTx-p. The vast majority of patients (around 90% at every time point) with low levels of 25-OH Vitamin D after RTx received supplemental therapy with cholecalciferol.

In Table 2 are listed the main biochemical parameters collected in our cohort.

**Table 2:** Main biochemical parameters at baseline and follow-up.

Variables	T1	T6	T12
Prot-U (g/24 h)	0.20 [0.14–0.30]	0.17 [0.11–0.26]	0.17 [0.11–0.25]
MDRD (ml/min/m^2^)	64.3 ± 26.8	63.0 ± 23.7	64.3 ± 23.6
Uric acid (mg/dl)	5.8 ± 1.6	6.5 ± 1.5	10.9 ± 1.4
Hemoglobin (g/dl)	10.9 ± 1.4	12.3 ± 1.5	12.8 ± 1.6
Albumin (g/dl)	4.1 ± 0.4	4.4 ± 0.4	4.4 ± 0.4
HbA1c (% of total Hb)	5.5 ± 0.7	5.8 ± 0.7	5.8 ± 0.7
CRP (mg/dl)	0.18 [0.08–0.56]	0.12 [0.07–0.3]	0.13 [0.07–0.34]
PTH (pg/ml)	86.8 ± 82	81.6 ± 79.6	77.5 ± 81
Corrected serum calcium (mg/dl)	9.7 ± 0.8	9.8 ± 0.7	9.8 ± 0.7
Serum phosphorus (mg/dl)	2.5 ± 0.9	3.1 ± 0.7	3.1 ± 0.6
Alcaline phosphatases (mg/dl)	103 ± 63	107 ± 76.8	94 ± 55
25-OH vitamin D (ng/dl)	12.6 [8.6–18.8]	14.2 [9.1–21.4]	17.1 [10.5–24.5]
1,25-OH vitamin D (pg/dl)	38.2 ± 23.8	49 ± 20.8	52.5 ± 11.6

T, month of follow-up; Prot-U, 24-h proteinuria collection; MDRD, modification of diet in renal disease; HbA1c, glycated haemoglobyin; CRP, C-reactive protein; PTH, parathormone.

Anthropometric and biochemical values are listed as mean ± standard deviation or median [interquartile range] as needed.

PTH levels were generally elevated at every time point, with mean 1st year PTH of 60.3 [40.4–93.6]. However a trend toward a decreasing pattern was observed during 1st year. Concerning the other MBD parameters, calcium and 1,25-OH Vitamin-D were normal in average, while 25-OH Vitamin D was markedly low, even if a progressive increase in its values during 1st year was observed.

### Risk factors for HPT development and HPT related disturbances during 1st year of RTx

As depicted in Table 3, we analyzed the eventual differences in PTH levels at different timepoints according to pre-RTx and baseline cohort characteristics. PTH levels were generally lower in pre-emptive patients and living donor graft was associated with markedly lower PTH levels at every timepoint. DGF correlated with significantly higher PTH levels especially at T1.

**Table 3:** Analysis of difference in PTH levels at different timepoints concerning baseline and pre-RTx patients’ characteristics.

Variables	PTH T1	PTH T6	PTH T12
Sex			
*M*	66 [40.6–106]	62.7 [40.6–98.9]	58.9 [37.4–91.2]
*F*	62.2 [37.1–101.9]	55.1 [37.5–88.7]	52.5 [36.7–81.7]
Dialysis type			
*Pre-emptive*	44.8 [29.5–74.5]	49 [30.9–80.5]	45 [28.1–78.3]
*PD*	61.7 [39.8–106.0]	54.6 [36.6–85.9]	55 [38–80.6]
*HD*	67.4 [40–108.3]^§§^	62.6 [40.5–99.3]^§§^	58.3 [38–90.9]^**^
Transplant donor			
*Cadaveric*	66.8 [41.9–110.1]	62.1 [40–98.8]	58.3 [39.7–91.6]
*Living*	48.3 [30.6–77.3]^##^	49 [35.8–79.8]^§§^	43.6 [30.9–74.3]^##^
Diabetes pre-RTx			
*Yes*	68 [39.4–100]	63.3 [39.8–100.9]	55.5 [36–89.3]
*No*	63.1 [37.6–105.7]	56.6 [38.1–94.4]	54.9 [36.5–87.6]
DGF			
*Yes*	83.4 [46.9–138.4]	67.0 [38.5–113.7]	63.8 [38–104.3]
*No*	61.2 [37.8–98.1]^§§^	57.6 [39.4–91.5]	55.3 [37.2–86.8]
Cinacalcet use before RTx			
*Yes*	101 [69.7–187.9]	110.3 [52.4–183.0]	105 [74.8–139.8]
*No*	65.7 [39.8–110.1]^§§^	57.1 [38.1–95.1]^##^	52.9 [36.5–83.4]^##^
Hyperparathyroidism type	T1	T6	T12
*SHPT*	71.9 [51.3–105.3]	64.2 [48.6–95.3]	64 [49.1–91.2]
*THPT*	116 [73.8–175.8]^##^	105.2 [74.3–154.3]^##^	97.8 [64.4–129.7]^##^
Vitamin D status	T1	T6	T12
*Deficient*	66.8 [39.6–110.1]	67.8 [45.8–106]	64.9 [42.7–103.5]
*Insufficient*	50.4 [33.5–79.5]	48.9 [36.4–66.7]	44.4 [31–68]
*Sufficient*	40.9 [25.4–66.8]^##^	36 [28.7–58.4]^##^	38.7 [28.7–53.3]^##^

RTx, renal transplantation; PTH, parathormone; HD, hemodialysis; PD, peritoneal dialysis; DGF, delayed graft function. PTH values are reported as median [interquartile range].

^**^p < 0.05; ^§§^*p* < 0.001; ^##^*p* < 0.0001.

Cinacalcet therapy before RTx strongly related to higher PTH levels post-RTx, at every time-point considered.

Most importantly THPT strongly correlated with higher PTH levels at every time point.

In Tables 4A, 4B the associations between the remaining anthropometric and biochemical factors and PTH levels during 1st year of RTx are shown.

**Table 4A:** Association between main anthropometric and biochemical factors at baseline and during 1st year of RTx with PTH levels, weighted for kidney function.

Variables	PTH T1 beta	*p*	PTH T6 beta	*p*	PTH T12 beta	*p*
Age at RTx (years)	0.035	0.40	−0.014	0.71	0.021	0.58
Dialysis vintage (months)	0.211	**<0.0001**	0.167	**<0.0001**	0.198	**<0.0001**
Cold ischemia time (hours)	−0.059	0.21	−0.068	0.148	−0.04	0.40
BMI (Kg/m^2^)						
*T1*	0.128	**0.001**	0.037	0.336	0.058	0.141
*T12*					0.047	0.229
Prot-U (g/24 h) **						
*T1*	0.072	**0.05**	0.046	0.23	0.032	0.40
*T6*			0.089	**0.020**	0.031	0.42
*T12*					0.013	0.73
MDRD (ml/min)						
*T1*	0.10	0.78	0.06	0.13	0.02	0.056
*T6*	0.008	0.83	−0.006	0.87	0.001	0.98
*T12*	−0.04		0.00	0.99	0.003	0.94
Hemoglobin (g/dl)**						
*T1*	−0.051	0.18	−0.023	0.54	−0.059	0.13
*T6*			0.023	0.54	0.021	0.58
*T12*					−0.024	0.54
HbA1c (% of total Hb)						
*T1*	−0.080	0.09	−0.080	0.10	−0.099	**0.04**
*T6*			−0.035	0.46	−0.108	**0.02**
*T12*					−0.097	**0.04**
Albumin (g/dl)						
*T1*	−0.086	**0.025**	−0.039	0.31	−0.051	0.19
*T6*			−0.128	**0.001**	−0.054	0.17
*T12*					−0.026	0.51
Uric Acid (mg/dl)						
*T1*	0.142	**0.001**	0.086	**0.041**	0.054	0.21
*T6*			0.159	**<0.0001**	0.164	**<0.0001**
*T12*					0.154	**<0.0001**
CRP (mg/dl)						
*T1*	−0.116	**0.004**	−0.100	**0.015**	−0.087	**0.036**
*T6*			−0.018	0.667	0.016	0.71
*T12*					0.093	**0.027**

Beta, linear regression analysis coefficient; RTx, renal transplantation; PTH, parathormone; T, month of follow-up; BMI, Body Mass Index; MDRD, modifcation of diet in renal disease; HbA1c, glycated hemoglobin; CRP, c-reactive protein. Bold values indicate significance at *p* values.

**Table 4B:** Association between main mineral metabolism parameters at baseline and during 1st year of RTx with PTH levels, weighted for kidney function.

Variables	PTH T1 beta	*p*	PTH T6 beta	*p*	PTH T12 beta	*p*
Corrected serum calcium (mg/dl)						
*T1*	0.272	**<0.0001**	0.379	**<0.0001**	0.275	**<0.0001**
*T6*			0.287	**<0.0001**	0.279	**<0.0001**
*T12*					0.198	**<0.0001**
Serum phosphorus (mg/dl)						
*T1*	−0.395	**<0.0001**	−0.421	**<0.0001**	−0.395	**<0.0001**
*T6*			−0.417	**<0.0001**	−0.385	**<0.0001**
*T12*					−0.398	**<0.0001**
Alcaline phosphatases (mg/dl)						
*T1*	0.368	**<0.0001**	0.270	**<0.0001**		**<0.0001**
*T6*			0.373	**<0.0001**		**<0.0001**
*T12*						**<0.0001**
25-OH vitamin D (ng/dl)						
*T1*	−0.255	**<0.0001**	−0.163	**<0.0001**	−0.214	**0.001**
*T6*			−0.350	**<0.0001**	−0.322	**<0.0001**
*T12*					−0.308	**<0.0001**
1,25-OH vitamin D (pg/ml)						
*T1*	0.164	**0.003**	0.040	0.49	0.011	0.85
*T6*			0.232	**<0.0001**	0.185	0.001
*T12*					0.280	**<0.0001**

Beta, linear regression analysis coefficient; RTx, renal transplantation; PTH, parathormone; T, month of follow-up. Bold values indicate significance at *p* values.

Dialysis vintage was directly associated with higher PTH levels at every time point.

eGFR, expressed with MDRD, did not show any significant association with PTH levels, and PTH did not appear to influence eGFR levels, during the first year. These results are also supported by further analyses reported in Supplementary Tables S4, S5. In Supplementary Table S4 is shown that with worsening CKD stage the proportion of patients with tertiary hyperparathyroidism significantly decreased at every time point considered. Moreover, there was no correlation between eGFR levels in different CKD stages and PTH levels, at every time point. So, hyperparathyroidism, especially tertiary hyperparathyroidism seemed to be associated with higher PTH levels and to be independent from eGFR during the first year of RTx. As for Prot-U any significant correlation with PTH was shown, apart from T6.

Uric acid and CRP were both directly associated with higher PTH levels at every time point. This relationship was particularly strong for uric acid, which was associated with higher PTH at all timepoints. Regarding mineral and bone metabolism parameters, the strongest association was found between 25-OH Vitamin D levels and PTH, with lower 25-OH Vitamin D levels associated with higher PTH levels. Moreover 25-OH Vitamin D status strongly correlated with higher PTH levels at every time point. 25-OH Vitamin D insufficiency was also correlated with significantly higher prevalence of THPT at every time point. On the contrary corrected serum calcium and alkaline phosphatase were associated with PTH in a direct proportionality fashion, as 1,25-OH Vitamin-D which was directly associated with HPT at T1 and T12.

At multivariate analyses, lower 25-OH Vitamin D, uric acid levels and cinacalcet therapy before RTx confirmed their independent association with HPT development (data not shown).

### Correlation of PTH with long term graft outcomes

**Figure 1:**
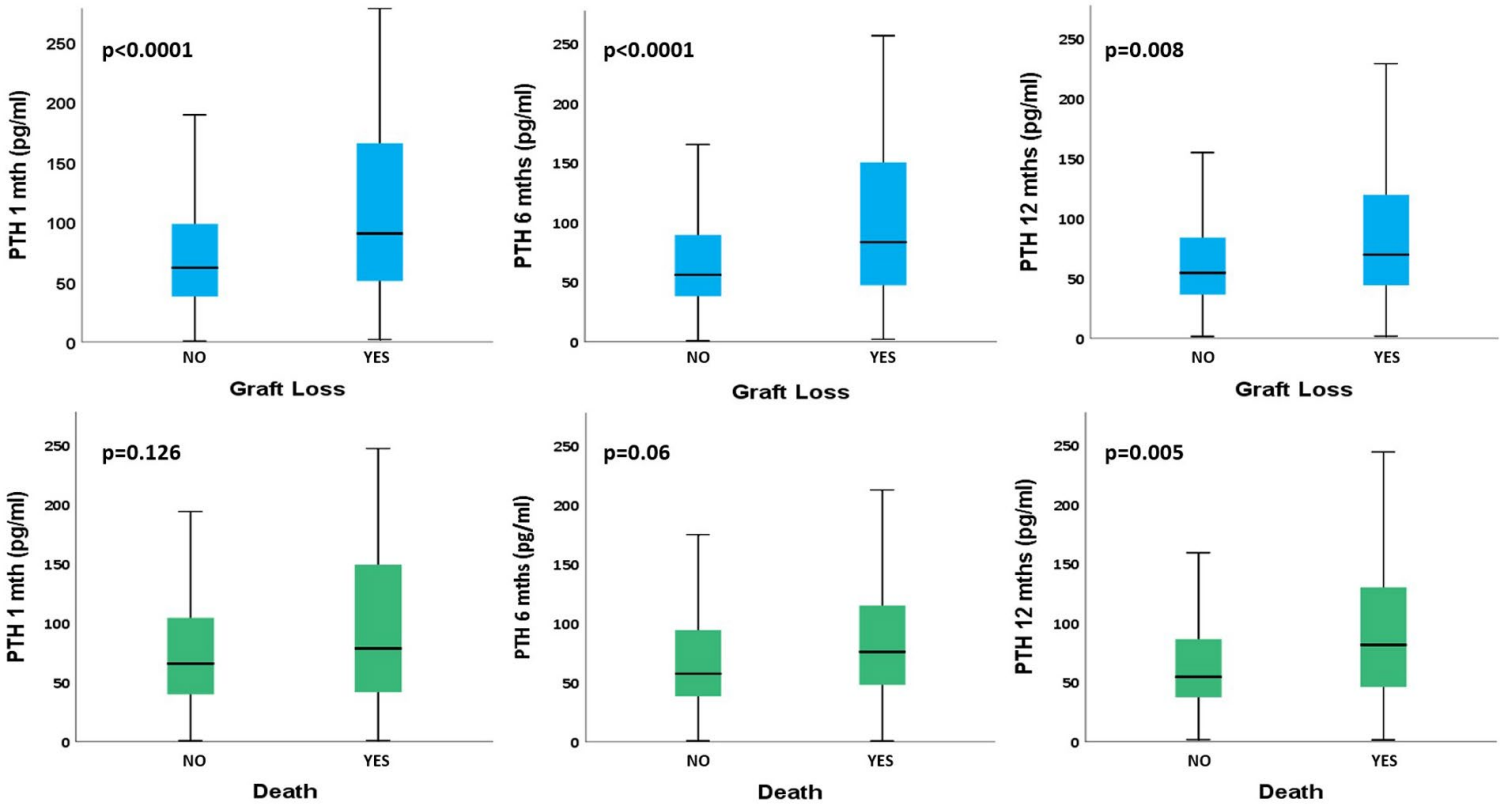
Comparison of PTH levels at different time points between different long-term patients’ outcomes. PTH, parathormone; T, month of follow-up. PTH values are reported as median [interquartile range].

We then evaluated the possible correlation between PTH levels during the 1st year after RTx and long-term graft outcome. As depicted in Figure 1 we found that PTH levels at all time points were strongly related to long term GL at T1 (GL+: 90.3 [50.6–165.6] vs. GL–: 61.7 [37.4–98.8], *p* < 0.0001), at T6 (GL+: 83.6 [47.1–152.4] vs. 56.1 [38.3–89.7], *p* < 0.0001) and T12 (GL+: 69.8 [44–121.9] vs. GL–: 54.6 [36.4–84.1], *p* = 0.008). The same trend of correlation was found between PTH and death for all causes, but a statistically significant relationship was found only at T12 (D+: 81.7 [45.4–130.8] vs. D–: 54.7 [37.4–86.8, *p* = 0.005]); detailed description of the data reported above is available on Supplementary Table S2.

Finally, we aimed to evaluate if HPT type might relate to long-term graft outcomes. In Supplementary Table S3 is shown that HPT in the first year after RTx, in particular THPT, strongly correlates with GL (T1: no HPT, 6% vs. SHPT, 11% vs. THPT 22%, *p* < 0.0001 – T6: no HPT, 9% vs. SHPT, 9% vs. THPT, 22%, *p* < 0.0001 – T12: no HPT, 7% vs. SHPT, 9% vs. THPT 21%, *p* < 0.001).

### PTH association with long-term graft loss

In our analyses, we aimed to evaluate the eventual independent association of PTH and HPT type with GL. Therefore, we built several multivariate models including in the first model type (*model 1*) T12 eGFR (through MDRD), T12 Prot-U, dialysis vintage, T12 Uric Acid along with mean 1st year PTH levels or HPT type at different time points. In the second model type (*model 2*) we included also graft rejection episodes during 1st year. The choice of these variables derived from preliminary analyses (not reported) testing which factors were more strongly associated with long-term GL.

As described in detail in Table 5, the main result was that mean 1st-year PTH was strongly and independently associated with long-term GL both in *model 1* and *model 2*.

**Table 5:** Association between 1st year HPT and long-term graft loss.

Dependent variable	Variables	Model 1		Model 2	
		OR	*p*	OR	*p*
Graft loss	*Mean-1st year PTH (pg/ml)*	2.62 (1.18–5.82)	**0.018**	2.82 (1.23–6.45)	**0.014**
	*THPT vs. No HPT (T1)*	2.63 (1.06–6.41)	**0.037**	2.60 (1.01–6.84)	**0.049**
	*THPT vs. SHPT (T1)*	2.5 (1.35–4.65)	**0.003**	2.24 (1.18–4.27)	**0.014**
	*THPT vs. No HPT (T6)*	2.04 (0.89–4.54)	0.094	1.83 (0.77–4.40)	0.172
	*THPT vs. SHPT (T6)*	2.81 (1.5–5.29)	**0.001**	2.1 (1.08–4.08)	**0.028**
	*THPT vs. No HPT (T12)*	2.38 (1.03–5.46)	**0.042**	2.27 (0.93–5.55)	0.061
	*THPT vs. SHPT (T12)*	2.60 (1.36–4.95)	**0.004**	2.23 (1.12–4.4)	**0.02**

HPT, hyperparathyroidism; T, month of follow-up; OR, odds ratio (multivariable logistic regression model); PTH, parathormone; THPT, tertiary hyperparathyroidism; SHPT, secondary hyperparathyroidism.

Model 1: model of analysis including dialysis vintage, T12 uric acid, T12 24-h proteinuria, renal function (MDRD) and mean 1st year PTH.

Model 2: model of analysis including dialysis vintage, T12 uric acid, T12 24-h proteinuria, renal function (MDRD), graft rejection during 1st year and mean 1st year PTH. Bold values indicate significance at *p* values.

Concerning THPT, stratified analyses were performed, comparing it first with HPT absence, and secondly with SHPT, in terms of long-term GL. In *model 1*, when compared to absence of HPT, THPT was independently associated to an increased odd of GL at T1 and T12. When *model 2* was performed, THPT was independently associated with GL only at T1. Instead, when compared to SHPT, THPT was independently associated with an increased risk of graft failure at every time-point considered, both in *model 1* and *model 2*.

### PTH reliability as marker of increased risk of long-term graft failure

**Figure 2:**
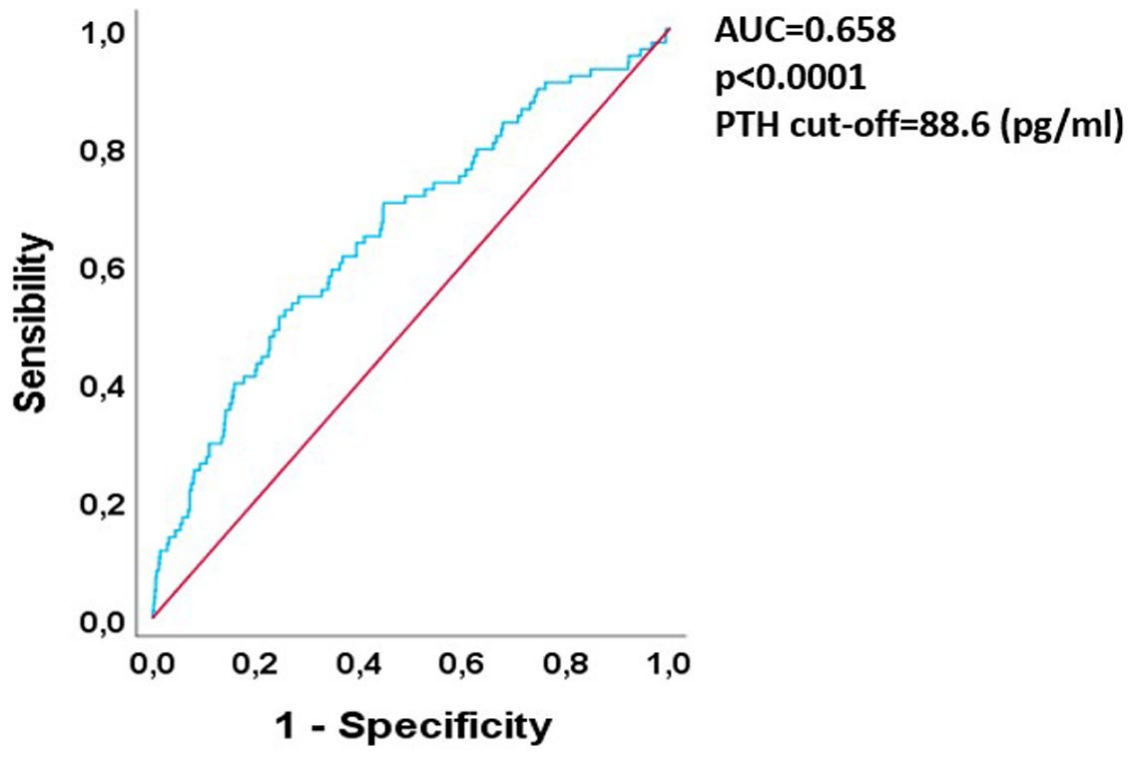
ROC curve analysis of 1st year PTH vs. long-term graft loss. ROC, receiver-operator curve; PTH, parathormone; AUC, area under curve.

We aimed at testing if mean PTH levels could have a predictive value concerning graft damage progression. So, we performed a ROC curve evaluating if a mean PTH cut-off for increased risk of long-term graft damage could be derived. The results are shown in Figure 2.

The curve showed a good predictive value for PTH in relationship to GL risk, since the area under curve (AUC) was 0.65 with a *p*-value < 0.0001. After finding the Youden index the best mean PTH cut off in terms of specificity was 88.6 pg/mL (specificity 0.75, LR+ 2.04, increase in the probability of long-term graft failure of 15% above this threshold).

### Impact of PTH and HPT type on long-term graft outcomes

**Figure 3:**
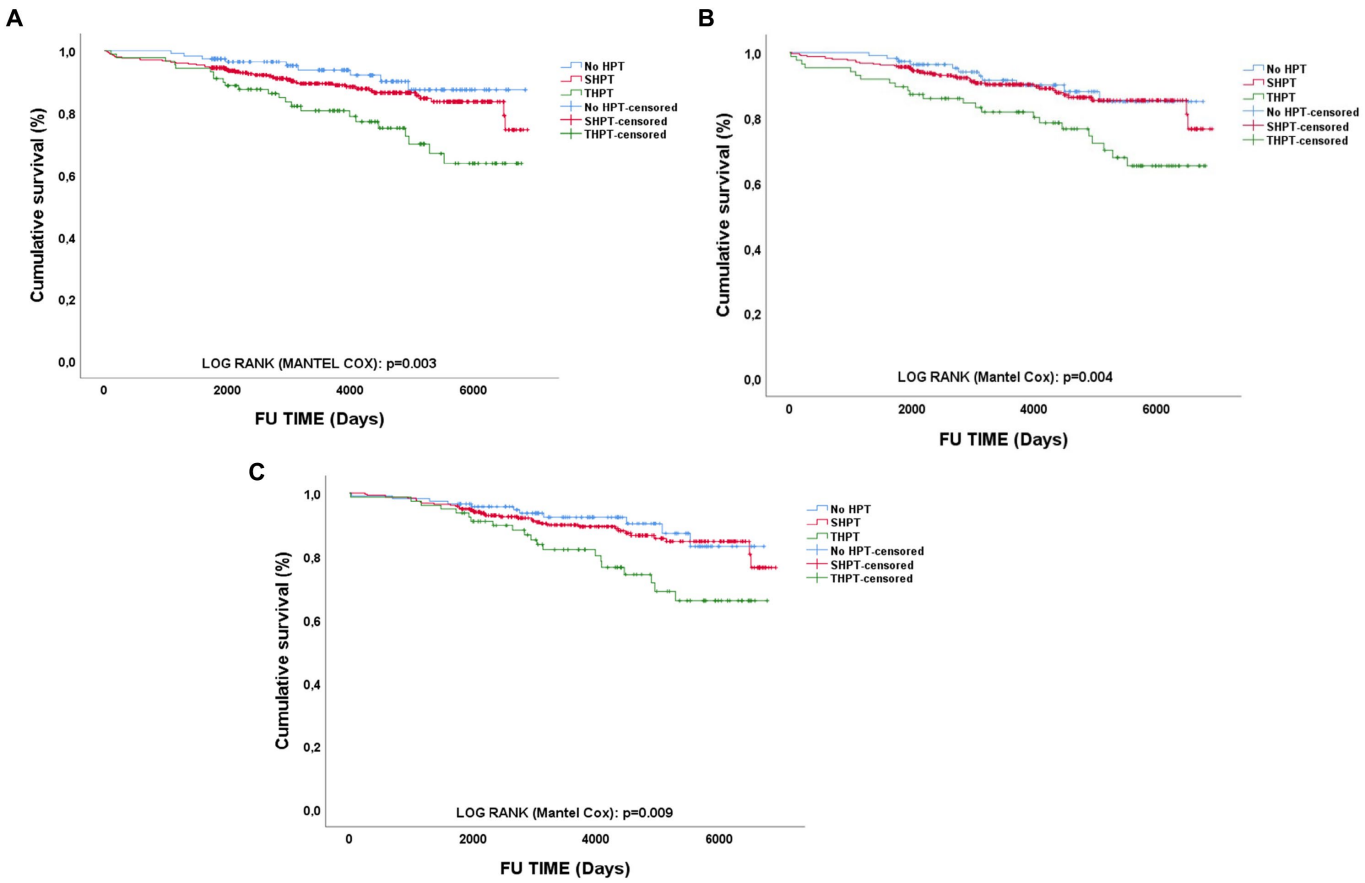
Survival analyses of long-term graft outcomes according to different PTH status. PTH type impact on long-term GL at different time points: **(A)** 1-month; **(B)** 6-months; **(C)** 12-months; HPT, hyperparathyroidism; SHPT, secondary hyperparathyroidism; THPT, tertiary hyperparathyroidism; FU, Follow-up.

As depicted in Figure 3, we performed survival analyses testing the influence of different HPT status during the 1st year of RTx on long-term GL. We found that, at every time point HPT, especially THPT, was strongly and significantly associated with worse long-term graft outcomes (Log-rank Mantel Cox T1, *p* = 0.003 – T6, *p* = 0.009 – T12, *p* = 0.004). In particular, in subgroups analyses no significant difference between HPT absence and SHPT was found at every time point. Instead, when comparing SHPT and THPT in terms of long-term GL, THPT was strongly and significantly associated with GL at every time point (Log-rank Mantel Cox T1, *p* = 0.011 – T6, *p* = 0.005 – T12, *p* = 0.009).

## Discussion

The main findings of our research were: (1) HPT has an important prevalence post-RTx. The most frequent form of HPT in the 1st year after RTx is SHPT, even if THPT has also a considerable prevalence. (2) Main risk factors in HPT development after RTx resulted pre-RTx Cinacalcet therapy, uric acid levels, hypovitaminosis-D. SHPT seems to be the one most associated with worse eGFR, while PTH levels and THPT seems to be independent from renal function during the 1st year. (3) High mean PTH levels during 1st year of RTx appear to be independently associated with long-term GL. Furthermore, mean PTH levels during the first year seems to have a good predictive power regarding long-term graft-loss. Of note, THPT seems to have the strongest association to graft damage development, especially when compared to SHPT. (4) The relationship between PTH, HPT and death for all-causes appeared to be less strong according to our analyses, therefore, more extensive analyses are needed to clarify these observations.

Our data are in accordance with many of the data present in the literature. Also, in our case the strongest risk factors for post-RTx HPT were longer dialysis vintage, pre-RTx Cinacalcet use, and lower 25-OH Vitamin-D levels (4–6, 20–22).

The apparent link between inflammatory and oxidative stress markers highlighted by our results tags along a rising body of evidence that links inflammation to HPT and Chronic Kidney Disease Mineral Bone Disorder (CKD-MBD) (23–30). On this matter, most interestingly, the relationship between Uric acid and high PTH levels is supported by some evidence in literature. In particular, a recent work performed on 8,316 patients, highlighted that, after adjusting for age, sex, dietary factors, glomerular filtration rate (GFR), and other potentially related biomarkers (calcium, phosphorus, alkaline-phosphatase, 25-hydroxyvitamin D), higher PTH seemed to be independently associated with higher uric acid levels. This relationship was particularly evident when considering patients at higher CKD stages (31).

In our work no significative difference in MDRD when compared with PTH levels and HPT status were found, during 1st year of RTx. The absence of difference in PTH levels according to baseline eGFR could be explained by the fact that in our cohort, mean baseline MDRD was above 60 mL/min, reducing the impact of renal function in determining PTH levels. In the study from Araujo et al. (11) persistent HPT was associated with lower MDRD at 6 months and at 1 year. The same observation was found also in the study from Isakov et al. (10) where eGFR was significantly lower 1 year after RTx and at 5 years-FU, but the threshold of PTH used for comparing patients was very high (>150 pg/mL). In both cases, this could have caused a bias, considering that very high PTH levels could be a direct consequence of a more severely impaired renal function at baseline, possibly weakening the significance of the association between PTH and graft function.

Regarding HPT impact on long-term GL many studies have obtained results that are similar to ours. Lou et al. found that treatment of HPT during 1st year of RTx was associated with reduced long-term GL. The same could be derived from the studies performed by Araujio and Isakov et al. as reported above (6, 10, 11). A recent work by Okada et al. performed in 892 RTx-p showed that death-censored graft survival after KTx was significantly lower in the normocalcemic-HPT group than in the HPT-free group. Cox hazard analysis also revealed that normocalcemic hyperparathyroidism was an independent risk factor for graft loss. Most importantly, the cohort characteristics were very similar to the one described in our study, especially in terms of HPT prevalence and FU time (32).

Differently from our study Pihlstrøm et al. found an association between HPT post-RTx and all-cause mortality. Interestingly, in this case, PTH levels were collected averagely at 5 years after RTx, suggesting that a prolonged exposure to high PTH levels could be the drive for an increased mortality rate (12). This observation is partially in accordance with our results. In fact, even if 1st year PTH was generally not associated to long-term mortality, the only significant correlation was found between PTH at 1 year and death for all causes.

Still there are some works that have found evidence in contrast with this hypothesis. Wolf et al. found no relationship between post-RTx HPT and long-term GL. However, in this case, PTH collection was not conducted at precise timepoints, but randomly during FU (8). Accordingly, Marcen et al. found no association between HPT during RTx and GL. Nevertheless, only univariate analyses were performed so the reported results were not adjusted for possible confounders (15).

Our study has some limitations. First, its retrospective design does not allow for a precise measure of risk association between PTH and graft outcome; Second complete data about patients’ pre-RTx status are lacking. This is due to the fact that the patients referred to our Transplant Center come from many different dialysis hubs from which it is difficult to collect data, especially in a standardized manner. Still data derived from our study, even if monocentric in nature, are collected from a really large cohort. Moreover, it has to be said that previous studies, of a similar nature and for number of patients, showed similar results. So, as said before, our study confirms what has been previously reported in the literature, but more detailed research is needed. Still, we managed to correct and test our observation for the main confounding factors in determining GL, so the strength of our evidence is strong. Another important factor in reducing biases of our observation is that PTH levels in our laboratory have been measured using the same validated method for many years. In future studies we aim to evaluate also the relationship between HPT and PTH levels with eGFR throughout all FU, as this would better elucidate and strengthen the associations reported. Moreover we also aim to evaluate the eventual relationship between HPT, bone-mineral density, CKD-MBD and overall graft outcome.

In conclusion, high PTH levels during first year of RTx were strongly associated to long-term GL. THPT was associated with increased risk of long-term GL when compared with HPT absence at T1 and T12, while it was associated with an increased risk of GL when compared to SHPT at every time point and after correction for all possible confounding factors. Moreover, in time-to-event analyses THPT was strongly associated with lower overall long-term graft survival at every time point considered.

## STROBE statement

The manuscript was checked to be compliant with STROBE checklist.

## Data availability statement

The raw data supporting the conclusions of this article will be made available by the authors, without undue reservation.

## Ethics statement

The studies involving human participants were reviewed and approved by Fondazione IRCCS Ca' Granda Ospedale Maggiore Policlinico Ethical Committee, Protocol ID 4759-1837/19. The patients/participants provided their written informed consent to participate in this study.

## Author contributions

CA, PaM, and GC: conceptualization. CA and PaM: methodology, formal analysis, and data curation. PaM: software and writing—original draft preparation. GC: validation, resources, and funding acquisition. PaM, EC, SV, MC, AR, DC, EF, LN, and EB: investigation. CA: writing—review and editing. CA and GC: supervision and project administration. PiM: supervision - methodology - validation. All authors have read and agreed to the published version of the manuscript.

## Conflict of interest

The authors declare that the research was conducted in the absence of any commercial or financial relationships that could be construed as a potential conflict of interest.

The handling editor VP declared a past collaboration (abstract presented during the ERA-EDTA 2022 congress) with the author CA.

## Publisher’s note

All claims expressed in this article are solely those of the authors and do not necessarily represent those of their affiliated organizations, or those of the publisher, the editors and the reviewers. Any product that may be evaluated in this article, or claim that may be made by its manufacturer, is not guaranteed or endorsed by the publisher.

## Glossary

AUC: Area Under Curve
BMI: Body Mass Index
CKD: Chronic Kidney Disease
CKD-MBD: Chronic Kidney Disease Mineral Bone Disorder
CPP: Calciprotein particles
CRP: C-reactive protein
D: Death
DGF: Delayed graft function
ECLIA: electro-chemiluminescence immune-assay
eGFR: estimated Glomerular Filtration Rate
ESRD: End Stage Renal Disease
FGF-23: Fibroblast Growth Factor 23
FU: Follow-up
GL: Graft Loss
HbA1c: Glycated Hemoglobin
HPT: Hyperparathyroidism
IL-6: Interleukin-6
LR: Likelihood Ratio
MBD: Mineral Bone Disorders
MDRD: Modification of Diet in Renal Disease
MM: Mineral Metabolism
Nf-Kb: Nuclear factor Kb
Prot-U: 24-h proteinuria
PTH: Parathormone
ROC: Receiver-Operator-Curve
RTx: Renal Transplant
RTx-p: Renal Transplant patients
sCr: serum creatinine
SHPT: Secondary Hyperparathyroidism
T: month of follow-up
THPT: Tertiary Hyperparathyroidism
VC: Vascular calcifications

## References

[1] Jørgensen HS Behets G Bammens B Claes K Meijers B Naesens M. Patterns of renal osteodystrophy 1 year after kidney transplantation. Nephrol Dial Transplant. (2021) 36:2130–9. doi: 10.1093/ndt/gfaB239, PMID: 34383929

[2] Evenepoel P Claes K Kuypers D Maes B Bammens B Vanrenterghem Y. Natural history of parathyroid function and calcium metabolism after kidney transplantation: a single-centre study. Nephrol Dial Transplant. (2004) 19:1281–7. doi: 10.1093/ndt/gfh128 14993493

[3] Torregrosa JV Fuster D Duran CE Oppenheimer F Muxí Á Rubello D. Set point of calcium in severe secondary hyperparathyroidism is altered and does not change after successful kidney transplantation. Endocrine. (2015) 48:709–11. doi: 10.1007/S12020-014-0312-0 24965230

[4] Messa P Sindici C Cannella G Miotti V Risaliti A Gropuzzo M. Persistent secondary hyperparathyroidism after renal transplantation. Kidney Int. (1998) 54:1704–13. doi: 10.1046/j.1523-1755.1998.00142.x 9844148

[5] Park JH Kang SW Jeong JJ Nam KH Chang HS Chung WY. Surgical treatment of tertiary hyperparathyroidism after renal transplantation: a 31-year experience in a single institution. Endocr J. (2011) 58:827–33. doi: 10.1507/endocrj.EJ11-0053, PMID: 21804261

[6] Lou I Foley D Odorico SK Leverson G Schneider DF Sippel R. How well does renal transplantation cure hyperparathyroidism? Ann Surg. (2015) 262:653–9. doi: 10.1097/SLA.0000000000001431, PMID: 26366545 PMC4576689

[7] Muirhead N Zaltman JS Gill JS Churchill DN Poulin-Costello M Mann V. Hypercalcemia in renal transplant patients: prevalence and management in Canadian transplant practice. Clin Transpl. (2014) 28:161–5. doi: 10.1111/ctr.12291, PMID: 24329899

[8] Wolf M Molnar MZ Amaral AP Czira ME Rudas A Ujszaszi A. Elevated fibroblast growth factor 23 is a risk factor for kidney transplant loss and mortality. J Am Soc Nephrol. (2011) 22:956–66. doi: 10.1681/ASN.2010080894, PMID: 21436289 PMC3083317

[9] Bleskestad IH Bergrem H Leivestad T Hartmann A Gøransson LG. Parathyroid hormone and clinical outcome in kidney transplant patients with optimal transplant function. Clin Transpl. (2014) 28:479–86. doi: 10.1111/ctr.12341 25649861

[10] Isakov O Ghinea R Beckerman P Mor E Riella LV Hod T. Early persistent hyperparathyroidism post-renal transplantation as a predictor of worse graft function and mortality after transplantation. Clin Transpl. (2020) 34:e14085. doi: 10.1111/ctr.14085 32949044

[11] Araujo MJCLN Ramalho JAM Elias RM Jorgetti V Nahas W Custodio M. Persistent hyperparathyroidism as a risk factor for long-term graft failure: the need to discuss indication for parathyroidectomy. Surgery. (2018) 163:1144–50. doi: 10.1016/j.surg.2017.12.010, PMID: 29331397

[12] Pihlstrøm H Dahle DO Mjøen G Pilz S März W Abedini S. Increased risk of all-cause mortality and renal graft loss in stable renal transplant recipients with hyperparathyroidism. Transplantation. (2015) 99:351–9. doi: 10.1097/tp.0000000000000583, PMID: 25594550

[13] Madorin C Owen RP Fraser WD Pellitteri PK Radbill B Rinaldo A. The surgical management of renal hyperparathyroidism. Eur Arch Otorhinolaryngol. (2012) 269:1565–76. doi: 10.1007/S00405-011-1833-2 22101574

[14] Kidney Disease: Improving Global Outcomes (KDIGO) CKD-MBD Work Group. KDIGO clinical practice guidelines for the diagnosis, evaluation, prevention, and treatment of chronic kidney disease-mineral and bone disorder (CKD-MBD). Kidney Int Suppl. (2009) 76:S1–S130. doi: 10.1038/ki.2009.188 19644521

[15] Marcén R Jimenez S Fernández A Galeano C Villafruela JJ Burgos FJ. The effects of mineral metabolism markers on renal transplant outcomes. Transplant Proc. (2012) 44:2567–9. doi: 10.1016/j.transproceed.2012.09.041 23146456

[16] Molinari P Alfieri CM Mattinzoli D Campise M Cervesato A Malvica S. Bone and mineral disorder in renal transplant patients: overview of pathology, clinical, and therapeutic aspects. Front Med. (2022) 9:821884. doi: 10.3389/fmed.2022.821884, PMID: 35360722 PMC8960161

[17] Messa P Alfieri CM. Secondary and tertiary hyperparathyroidism. Front Horm Res. (2019) 51:91–108. doi: 10.1159/000491041 30641516

[18] Froissart M Rossert J Jacquot C Paillard M Houillier P. Predictive performance of the modification of diet in renal disease and cockcroft-gault equations for estimating renal function. J Am Soc Nephrol. (2005) 16:763–73. doi: 10.1681/ASN.2004070549 15659562

[19] Masson I Flamant M Maillard N Rule AD Vrtovsnik F Peraldi MN. MDRD versus CKD-EPI equation to estimate glomerular filtration rate in kidney transplant recipients. Transplantation. (2013) 95:1211–7. doi: 10.1097/TP.0b013e318288caa6, PMID: 23511243

[20] Lorenz K Bartsch DK Sancho JJ Guigard S Triponez F. Surgical management of secondary hyperparathyroidism in chronic kidney disease--a consensus report of the European Society of Endocrine Surgeons. Langenbeck’s Arch Surg. (2015) 400:907–27. doi: 10.1007/S00423-015-1344-5 26429790

[21] Sutton W Chen X Patel P Karzai S Prescott JD Segev DL. Prevalence and risk factors for tertiary hyperparathyroidism in kidney transplant recipients. Surgery. (2022) 171:69–76. doi: 10.1016/j.surg.2021.03.067, PMID: 34266650 PMC8688275

[22] Evenepoel P Claes K Kuypers DR Debruyne F Vanrenterghem Y. Parathyroidectomy after successful kidney transplantation: a single centre study. Nephrol Dial Transplant. (2007) 22:1730–7. doi: 10.1093/ndt/gfm044 17371780

[23] Yamada S Tsuruya K Kitazono T Nakano T. Emerging cross-talks between chronic kidney disease-mineral and bone disorder (CKD-MBD) and malnutrition-inflammation complex syndrome (MICS) in patients receiving dialysis. Clin Exp Nephrol. (2022) 26:613–29. doi: 10.1007/S10157-022-02216-X 35353283 PMC9203392

[24] Komaba H. Energy sensor as a new regulator of FGF23 synthesis. Kidney Int. (2018) 94:453–5. doi: 10.1016/j.kint.2018.05.008 30143064

[25] Vidal A Rios R Pineda C Lopez I Muñoz-Castañeda JR Rodriguez M. Direct regulation of fibroblast growth factor 23 by energy intake through mTOR. Sci Rep. (2020) 10:1795. doi: 10.1038/S41598-020-58663-7, PMID: 32020002 PMC7000745

[26] Glosse P Feger M Mutig K Chen H Hirche F Hasan AA. AMP-activated kinase is a regulator of fibroblast growth factor 23 production. Kidney Int. (2018) 94:491–501. doi: 10.1016/j.kint.2018.03.006, PMID: 29861059

[27] Singh S Grabner A Yanucil C Schramm K Czaya B Krick S. Fibroblast growth factor 23 directly targets hepatocytes to promote inflammation in chronic kidney disease. Kidney Int. (2016) 90:985–96. doi: 10.1016/j.kint.2016.05.019, PMID: 27457912 PMC5065745

[28] Munoz Mendoza J Isakova T Ricardo AC Xie H Navaneethan SD Anderson AH. Fibroblast growth factor 23 and inflammation in CKD. Clin J Am Soc Nephrol. (2012) 7:1155–62. doi: 10.2215/CJN.13281211, PMID: 22554719 PMC3386678

[29] Komaba H Zhao J Yamamoto S Nomura T Fuller DS McCullough KP. Secondary hyperparathyroidism, weight loss, and longer term mortality in haemodialysis patients: results from the DOPPS. J Cachexia Sarcopenia Muscle. (2021) 12:855–65. doi: 10.1002/JCSM.12722, PMID: 34060245 PMC8350219

[30] Kir S Komaba H Garcia AP Economopoulos KP Liu W Lanske B. PTH/PTHrP receptor mediates cachexia in models of kidney failure and cancer. Cell Metab. (2016) 23:315–23. doi: 10.1016/j.cmet.2015.11.003, PMID: 26669699 PMC4749423

[31] Hui JY Choi JWJ Mount DB Zhu Y Zhang Y Choi HK. The independent association between parathyroid hormone levels and hyperuricemia: a national population study. Arthritis Res Ther. (2012) 14:R56. doi: 10.1186/AR3769 22405053 PMC3446422

[32] Okada M Tominaga Y Sato T Tomosugi T Futamura K Hiramitsu T. Elevated parathyroid hormone one year after kidney transplantation is an independent risk factor for graft loss even without hypercalcemia. BMC Nephrol. (2022) 23:212. doi: 10.1186/S12882-022-02840-5, PMID: 35710357 PMC9205154

