# Corrigendum: Impact of hyperparathyroidism and its different subtypes on long term graft outcome: a single Transplant Center cohort study

**DOI:** 10.3389/fmed.2024.1362741

**Published:** 2024-01-30

**Authors:** Paolo Molinari, Anna Regalia, Alessandro Leoni, Mariarosaria Campise, Donata Cresseri, Elisa Cicero, Simone Vettoretti, Luca Nardelli, Emilietta Brigati, Evaldo Favi, Piergiorgio Messa, Giuseppe Castellano, Carlo M. Alfieri

**Affiliations:** ^1^Unit of Nephrology, Dialysis and Kidney Transplantation, Fondazione IRCCS Ca' Granda Ospedale Maggiore Policlinico di Milano, Milan, Italy; ^2^Post-Graduate School of Specialization in Nephrology, University of Milano, Milan, Italy; ^3^Department of Clinical Sciences and Community Health, Università degli Studi di Milano, Milan, Italy; ^4^General Surgery and Kidney Transplantation, Fondazione IRCCS Ca' Granda Ospedale Maggiore Policlinico, Milan, Italy; ^5^Department of Nephrology, Dialysis and Kidney Transplants, IRCCS Ca' Granda Foundation Maggiore Policlinico Hospital, Milan, Italy

**Keywords:** kidney transplantation, parathormone, hyperparathyroidism, tertiary hyperparathyroidism, graft outcome

In the published article, an author name was incorrectly written as “Emilietta Brigatti”. The correct spelling is “Emilietta Brigati”.

The authors apologize for this error and state that this does not change the scientific conclusions of the article in any way. The original article has been updated.

