# Supplementary material for: Impact of hyperparathyroidism and its different subtypes on long term graft outcome: a single Transplant Center cohort study
Source: Front Med (Lausanne). 2023 Aug 10;10:1221086. doi: 10.3389/fmed.2023.1221086 (PMC10449540; doi:10.3389/fmed.2023.1221086)
Supplement: Supplementary file 1 [file Table_1.DOCX]

**Table S1:** MBD related therapies at baseline and follow-up.

| **Cinacalcet use before RTx**  **Yes**  **No** | 24 (5)  424 (94) |
| --- | --- |
| **Vitamin D Supplementation T1**  **No**  **Native Vitamin D**  **Active Vitamin D** | 688 (83)  73 (9)  65 (8) |
| **Vitamin D Supplementation T6**  **No**  **Native Vitamin D**  **Active Vitamin D** | 391 (74)  60 (11)  77 (15) |
| **Vitamin D Supplementation T12**  **No**  **Native Vitamin D**  **Active Vitamin D** | 537 (68)  152 (19)  95 (12) |
| **Cinacalcet T1**  **Yes**  **No** | 16 (3)  600 (97) |
| **Cinacalcet T6**  **Yes**  **No** | 28 (5)  579 (95) |
| **Cinacalcet T12**  **Yes**  **No** | 36 (6)  564 (94) |

**Note:** T: month of follow-up; HPT: hyperparathyroidism; MBD; mineral-bone disease; RTx: renal transplantation.

Numerosity is reported as number (percentage).

**Table S2**: Comparison of PTH levels at different timepoints according to GL and death for all-cause.

| **Variables** | **PTH T1** | **PTH T6** | **PTH T12** |
| --- | --- | --- | --- |
| **Graft Loss**  **Yes**  **No** | 90.3 [50.6-165.6]  61.7 [37.4-98.8] | 83.6 [47.1-152.4]  56.1 [38.3-89.7] | 69.8 [44-121.9]  54.6 [36.4-84.1] |
| **Death**  **Yes**  **No** | 78.6 [40.5-155.5]  65.7 [39.8-104.5] | 75.9 [47.2-117]  57.5 [38.3-94.4] | 81.7 [45.4-130.8]  54.7 [37.4-86.8] |

**Note:** T: month of follow-up; PTH: parathormone; GL: graft loss

Data are reported as median [interquartile range].

**Table S3:** Correlation between HPT type at different time points and long term graft outcomes.

| **Variables** | **HPT T1** | | | **HPT T6** | | | **HPT T12** | | |
| --- | --- | --- | --- | --- | --- | --- | --- | --- | --- |
|  | **No** | **Second.** | **Tert.** | **No** | **Second.** | **Tert.** | **No** | **Second.** | **Tert.** |
| **Graft loss**  **Yes**  **No** | 9 (6)  144 (94) | 47 (11)  401 (89) | 24 (22) ##  88 (78) | 12 (9)  128 (91) | 41 (9)  411 (91) | 24 (22) ##  87 (78) | 12 (7)  147 (93) | 39 (9)  381 (91) | 21 (21) §§  78 (79) |
| **Death**  **Yes**  **No** | 11 (9)  115 (91) | 42 (11)  345 (89) | 12 (12)  89 (88) | 11 (9)  112 (91) | 39 (10)  343 (90) | 16 (16)  86 (84) | 11 (8)  120 (92) | 34 (9)  323 (91) | 12 (13)  80 (87) |

**Note:** HPT: hyperparathyroidism; T: month of follow-up. ##: p<0.001; §§: p<0.0001

Numerosity is reported as number (percentage).

**Table S4:** Correlation of CKD stages at different timepoints with hyperparathyroidism.

| **Variables** | **HPT T1** | | | **HPT T6** | | | **HPT T12** | | |
| --- | --- | --- | --- | --- | --- | --- | --- | --- | --- |
|  | **No** | **Second.** | **Tert.** | **No** | **Second.** | **Tert.** | **No** | **Second.** | **Tert.** |
| **CKD STAGE T1**  **1**  **2**  **3a**  **3b**  **4** | 27 (23)  55 (23)  45 (24)  19 (14)  5 (21) | 64 (55)  147 (60)  118 (63)  98 (71)  18 (75) | 25 (22)  41 (17)  24 (13)  21 (15)  1 (4) | 19 (17)  54 (23)  39 (20)  24 (18)  4 (16) | 67 (60)  135 (57)  132 (68)  94 (70)  20 (80) | 25 (22)  46 (20)  22 (11)  16 (20)  1 (4)^##^ | 29 (17)  58 (20)  36 (12)  27 (10)  8 (4) | 62 (56)  124 (54)  119 (68)  93 (70)  19 (68) | 19 (17)  45 (20)  21 (12)  13(10)  1 (4)^##^ |
| **CKD STAGE T6**  **1**  **2**  **3a**  **3b**  **4** |  |  |  | 16 (17)  56 (23)  44 (21)  23(17)  1 (6) | 58 (62)  140 (58)  133 (64)  102 (75)  15 (83) | 19 (20)  46 (19)  32 (15)  11 (8)  2 (11)^##^ | 19 (22)  62 (25)  47 (23)  28 (22)  1 (6) | 47 (55)  139 (57)  131 (65)  87 (68)  15 (94) | 20 (23)  43 (18)  22 (11)  13 (10)  0 (0)^##^ |
| **CKD STAGE T12**  **1**  **2**  **3a**  **3b**  **4** |  |  |  |  |  |  | 18 (21)  70 (27)  41 (22)  26 (21)  2 (13) | 47 (55)  149 (57)  120 (63)  92 (74)  12 (80) | 20 (23)  42 (16)  29 (15)  7 (6)  1 (7)^$$^ |

**Note:** CKD: chronic kidney disease; T: month of follow-up; HPT: hyperparathyroidism.

**Table S5:** Linear regression analyses between eGFR in different CKD stages and PTH levels.

| **CKD STAGE** | **PTH T1**  **Beta** | **p** | **PTH T6**  **Beta** | **p** | **PTH T12**  **Beta** | **p** |
| --- | --- | --- | --- | --- | --- | --- |
| **CKD STAGE 1**  **T1**  **T6**  **T12** | 0.000 | 0.998 | 0.000  0.000 | 0.957  0.998 | -0.062  0.041  0.072 | 0.523  0.711  0.515 |
| **CKD STAGE 2**  **T1**  **T6**  **T12** | -0.031 | 0.636 | -0.012  -0.044 | 0.850  0.498 | -0.018  0.019  0.010 | 0.672  0.770  0.872 |
| **CKD STAGE 3a**  **T1**  **T6**  **T12** | -0.094 | 0.443 | 0.005  -0.035 | 0.940  0.617 | -0.019  0.036  -0.080 | 0.799  0.611  0.274 |
| **CKD STAGE 3b**  **T1**  **T6**  **T12** | 0.008 | 0.927 | -0.039  -0.080 | 0.658  0.353 | -0.072  0.015  -0.048 | 0.407  0.869  0.594 |
| **CKD STAGE 4**  **T1**  **T6**  **T12** | -0.193 | 0.367 | -0.118  **-0.444** | 0.573  **0.03** | -0.048  **-0.335**  -0.250 | 0.810  **0.04**  0.235 |

**Note:** CKD: chronic kidney disease; T: month of follow-up; HPT: hyperparathyroidism
